# Optimal Reference Genes for Gene Expression Normalization in *Trichomonas vaginalis*

**DOI:** 10.1371/journal.pone.0138331

**Published:** 2015-09-22

**Authors:** Odelta dos Santos, Graziela de Vargas Rigo, Amanda Piccoli Frasson, Alexandre José Macedo, Tiana Tasca

**Affiliations:** 1 Faculdade de Farmácia, Universidade Federal do Rio Grande do Sul, Porto Alegre, RS, Brazil; 2 Centro de Biotecnologia, Universidade Federal do Rio Grande do Sul, Porto Alegre, RS, Brazil; Northwestern University, UNITED STATES

## Abstract

*Trichomonas vaginalis* is the etiologic agent of trichomonosis, the most common non-viral sexually transmitted disease worldwide. This infection is associated with several health consequences, including cervical and prostate cancers and HIV acquisition. Gene expression analysis has been facilitated because of available genome sequences and large-scale transcriptomes in *T*. *vaginalis*, particularly using quantitative real-time polymerase chain reaction (qRT-PCR), one of the most used methods for molecular studies. Reference genes for normalization are crucial to ensure the accuracy of this method. However, to the best of our knowledge, a systematic validation of reference genes has not been performed for *T*. *vaginalis*. In this study, the transcripts of nine candidate reference genes were quantified using qRT-PCR under different cultivation conditions, and the stability of these genes was compared using the geNorm and NormFinder algorithms. The most stable reference genes were *α-tubulin*, *actin* and *DNATopII*, and, conversely, the widely used *T*. *vaginalis* reference genes *GAPDH* and *β-tubulin* were less stable. The *PFOR* gene was used to validate the reliability of the use of these candidate reference genes. As expected, the *PFOR* gene was upregulated when the trophozoites were cultivated with ferrous ammonium sulfate when the *DNATopII*, *α-tubulin* and *actin* genes were used as normalizing gene. By contrast, the *PFOR* gene was downregulated when the *GAPDH* gene was used as an internal control, leading to misinterpretation of the data. These results provide an important starting point for reference gene selection and gene expression analysis with qRT-PCR studies of *T*. *vaginalis*.

## Introduction

The most common non-viral sexually transmitted disease (STD) worldwide is trichomonosis that is caused by *Trichomonas vaginalis*, which is a flagellate parasitic protozoan, with an incidence estimated of 276.4 million new cases each year [1]. Recently, *T*. *vaginalis* was classified among five parasitic infection neglected in the United States of America [2]. Serious health consequences have been associated with trichomonosis such as infertility, predisposition to cervical cancer, pelvic inflammatory disease and adverse pregnancy outcomes like low birth weight babies and preterm birth [3,4]. In addition, aggressive prostate cancers have been associated with trichomonosis [5]. Other important aspect about the trichomonosis is a positive association between *T*. *vaginalis* infection and human immunodeficiency virus (HIV) transmission [6]. The complete genome from *T*. *vaginalis* was published in 2007 [7], consequently this could allow greatly progress in pathogenicity studies, as it has occurred with other organisms when the complete genomes and transcriptomes sequences were available [8,9]. For these approaches, the measurement of gene expression using quantitative real-time transcriptase reverse PCR (qRT-PCR) is chosen because it is rapid, sensitive and precise particularly to detect a few copies from mRNA [8, 10–12]. However, data analysis could have errors due to technical and experimental variability [13]. Therefore, to avoid the effect of these factors, the use of housekeeping genes is necessary [13, 14]. The low or absent variability in its expression level is expected for one reference gene when exposed to different types of experimental treatments [13, 15, 16]. However, frequently used housekeeping genes such as *glyceraldehyde-3-phosphate* (*GAPDH*), *elongation factor* and *β-actin*, have displayed unstable expression levels under different experimental studies, therefore demonstrating the importance of establishing a set of optimal reference genes, before starting a gene expression analysis [13,17,18]. Nevertheless, there are no reported studies of the validation of reference genes in *T*. *vaginalis*, and genes such as *α-* or *β-tubulin* are frequently used as housekeeping genes for normalization in *T*. *vaginalis* gene expression analyses [19,20]. In this study, nine candidate reference genes previously used in studies with *T*. *vaginalis* or other eukaryotes, were selected and their mRNA levels were measured in trichomonads under nutrient-deficient conditions and high iron conditions by qRT-PCR. Next, two algorithms NormFinder and geNorm were used to classify the suitable reference genes for the normalization of gene expression in *T*. *vaginalis* [15, 21]. To confirm the consistency of the selected reference genes, pyruvate:ferredoxin oxidoreductase (*PFOR*) was used as the target gene. *PFOR* is a hydrogenosomal enzyme that has homology with the 120 kDa surface glycoprotein (AP120) involved in the *T*. *vaginalis* cytoadherence process, and it is up-regulated in the presence of iron [22]. Therefore, the three most stable and one unstable candidate reference genes were selected for testing for normalization of the relative expression of *PFOR* in trichomonads cultivated under high iron concentrations. The results obtained here represent important information for reference gene selection to use in gene expression studies in *T*. *vaginalis*.

## Results

### Selection of candidate RT-qPCR reference genes for *Trichomonas vaginalis*

To identify a set of potential reference genes, a thorough review of the scientific literature with a focus on genes traditionally used as normalizers for gene expression in *T*. *vaginalis* studies was performed. Of five reference genes often used for expression assays: *β-tubulin* [19,22,23,24,25,26,27,28,29,30,31,32], *α-tubulin* [33,34,35,36,37,38], *60S rRNA* [39,40,41], *GAPDH* [38,42], and *coronin* [8], the three most used genes for normalization were selected, *β-tubulin*, *α-tubulin*, and *coronin*. An additional six candidate genes were included because of their use as the best reference genes for other organisms, and a total of nine candidate genes were selected, *actin*, *F-actin (β* and *α)*, *tubulin (α*, *β* and *γ)*, *glyceraldehyde 3-phosphate dehydrogenase (GAPDH)*, *elongation factor (Efa)*, and *DNA topoisomerase II (DNATopII)* (Table 1). The nucleotide sequences of all candidate reference genes were obtained from the *T*. *vaginalis* genome project database (TrichDB http://trichdb.org/trichdb/), where more than one sequence was found. To select only one nucleotide sequence for each gene, the following approach was used: all sequences obtained with a score equal to 100 in the TrichDB search were aligned using the ClustalW2 –Multiple Sequence Alignment (http://www.ebi.ac.uk/Tools/msa/clustalw2/), and the phylogenetic analysis was performed using MEGA (Molecular Evolutionary Genetics Analysis software) (S1 Fig). Next, only one nucleotide sequence for each gene was selected from the branch subtypes with a greater number of individuals. The primer pairs specificities were verified with a BLAST (Basic Local Alignment Search Tool; at http://blast.ncbi.nlm.nih.gov/) search and the primer pairs designed were used only if they did not amplify human or other *Trichomonas* species sequences.

**Table 1:** Gene ID, gene symbol, primer sequence and amplicon length of the selected reference genes.

Gene ID	Gene Symbol	Primer sequence (5’ to 3’)	Amplicon length (bp)
TVAG_534990	*Actin*	F: TCACAGCTCTTGCTCCACCA	175
		R: AAGCACTTGCGGTGAACGAT	
TVAG_212270	*F-actin α*	F: ATCGACGAAGGCATCAAAGC	103
		R: TACGAGCTTCCTCGCAAAGG	
TVAG_271840	*F-actin β*	F: TCTTCGGATGCGGTGTTTTC	178
		R: CCGATTCCAACGTCAAGCTC	
TVAG_206890	*α*-*tubulin*	F: TGCCCAACAGGCTTCAAGAT	101
		R: TTAGCGAGCATGCAGACAGC	
TVAG_073800	*β-tubulin*	F: TCCGTGGCCGTATGTCATCT	169
		R: GCTGTTGTGTTGCCGATGAA	
TVAG_109820	*γ-tubulin*	F: TGCCGATGCTCTTGAAGGAT	173
		R: TGTATGGGGCAACAACGACA	
TVAG_03880	*DNATopII*	F: ATCGGTGTCGGTTGGTCAAG	171
		R: TGGCTGTTTGACACCGTCTTT	
TVAG_067400	*Efa*	F: CACAACAACAGGCCACCTCA	136
		R: TTCAGCCTTGAGGGAGTCCA	
TVAG_475220	*GAPDH*	F: GCCGCAAGCTCTATCCAAAG	196
		R: CGGCCACCGATTGACTTAAC	

### PCR Amplification Specificity and PCR efficiency

The specific melting temperature, corresponding to a single peak was found for all nine candidate reference genes (S2A Fig and Table 2) and a single band for each product was visualized in the agarose gel electrophoresis (S2B Fig), indicating the specificity of all primer pairs used for qRT-PCR. The DNA calibration curves of ten-fold dilution series were used to calculate the regression coefficient (R^2^) for each candidate reference gene, thus the primer efficiency was evaluated. The PCR efficiency range from 90% for *DNATopII β* to 110% for *γ-tubulin* is shown in Table 2. No primer dimers or unexpected amplicons were observed from non-specific amplification, and no signals were detected in the negative controls.

**Table 2:** Results from standard curves of the selected candidate references genes: slopes, amplification efficiency (E), annealing temperature (Ta), melting temperature (Tm) and primer concentration.

Gene Symbol	Slope	E (%)	R^2^	Ta (°C)	Tm (°C)	Primer concentration (μM)
*Actin*	-3.527	92	0.990	64	86.3	0.1
*F-actin α*	-3.318	100	0.986	64	81.5	0.1
*F-actin β*	-3.551	91	0.978	63	83.3	0.1
*α*-*tubulin*	-3.472	94	0.997	64	86.3	0.2
*β-tubulin*	-3.452	95	0.991	64	84.7	0.1
*γ-tubulin*	-2.787	110	0.994	64	80.3	0.2
*DNATopII*	-3.329	90	0.993	64	81.8	0.1
*Efa*	-3.504	99	0.996	64	84.8	0.2
*GAPDH*	-3.316	100	0.992	64	84.8	0.2

### Expression levels of the selected candidate reference genes

To recognize the ideal reference genes for normalization of the gene expression profiles in *T*. *vaginalis*, the raw Ct values were used to measure the expression stabilities of the nine candidate genes. All data were examined under the following five subsets: (1) *T*. *vaginalis* grown in trypticase-yeast extract-maltose (TYM) medium supplemented with 10% heat-inactivated bovine serum (HIBS) (2); HIBS restriction (1.0%) (3); maltose restriction (273 μM); (4) HIBS and maltose restriction; and (5) TYM supplemented with 200 μM ferrous ammonium sulfate.

**Fig 1:**
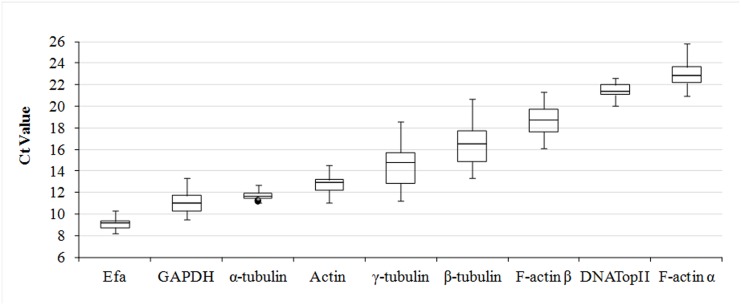
Raw Ct values from qRT-PCR of candidate reference genes under all cultivation conditions. Box shows the 25/75 percentiles and the *whiskers* indicate the maximum and minimum values. The median is presented by the line and outliers are exhibited by dots.

The expression levels of the nine reference genes for all sets of samples are presented in Fig 1 using the raw Ct values. Diverse levels of mRNA copy were observed for these genes, with the Ct values ranging from 8 cycles in *Efa* gene to 25 cycles for the *F-actin α* gene. A constant expression level was not found for none of the tested reference gene in all conditions tested. The dispersion of the Ct values was the lowest for the *Efa*, *α-tubulin*, *actin*, and *DNATopII* genes, indicating the lowest gene expression variations, whereas the *γ-tubulin* and *β-tubulin* genes showed the highest variability in the CT value, and consequently, the highest gene expression variations. Five candidate reference genes had average Ct values below 15 cycles, including *Efa*, *GAPDH*, *α-tubulin*, *actin*, and *γ-tubulin*, indicating higher expression levels. By contrast, *F-actin α*, *DNATopII*, *F-actin β*, and *β-tubulin* had average Ct values above 15, indicating that these genes produced fewer transcripts. Consequently, the *Efa* gene presented the highest abundant expression level, whereas the *F-actin α* gene had the lowest level.

The coefficient of variation (CV%) values were calculated for each candidate reference gene after grouping the samples into the following three different sets: (i) total samples (all growth conditions); (ii) all samples subjected to nutrient restriction; and (iii) samples cultivated with ferrous ammonium sulfate. The CV was calculated as CV = σ/μ, where σ is the standard deviation of the Ct values of a candidate reference gene and μ is the Ct value mean for the same gene. These values were also evaluated to verify the expression stability for each candidate gene. CV values lower than 4.0% were obtained for *DNATopII* when all samples were analyzed and when the samples from the nutrient restriction were grouped into one set (Table 3). Of the other potential reference genes, CVs lower than 4.0% were obtained for *actin* and *F-actin α* when the parasites were cultivated with ferrous ammonium sulfate. Conversely, *β-tubulin*, *GAPDH*, and *γ-tubulin* presented high CV values under the three conditions sets, with CV values ranging from 9.9% to 40.3% (Table 3).

**Table 3:** The coefficient of variation (CV %) of candidate reference genes from *T*. *vaginalis* under three distinct sets.

Gene	Total samples	Gene	All nutrient restriction	Gene	Ferrous ammonium sulfate
	CV %		CV %		CV %
*DNATopII*	2.9	*DNATopII*	2.42	*Actin*	2.5
*F-actin α*	4.4	*α-tubulin*	4.5	*F-actin α*	3.8
*α-tubulin*	4.7	*F-actin α*	4.9	*F-actin β*	4.4
*Efa*	5.0	*Actin*	5.2	*α-tubulin*	4.7
*Actin*	6.2	*Efa*	5.7	*DNATopII*	7.6
*F-actin β*	6.3	*F-actin β*	6.4	*Efa*	11.9
*β-tubulin*	10.0	*β-tubulin*	9.9	*β-tubulin*	39.2
*GAPDH*	10.1	*GAPDH*	10.3	*γ-tubulin*	39.6
*γ-tubulin*	13.4	*γ-tubulin*	13.2	*GAPDH*	40.3

### Expression stability of the candidate reference genes

Two different statistical algorithms NormFinder and geNorm were used to rank the candidate reference genes according to their expression stability [43].

#### geNorm analysis

The geNorm was used to calculate the expression stability value (*M*) for each candidate reference gene and the pairwise variation (*V*) of a certain gene compared with others. To do the analysis using the geNorm software the raw Ct values were grouped into the following six experimental datasets: (i) total samples; (ii) all samples under nutrient restriction; (iii) samples under HIBS restriction; (iv) samples under maltose restriction; (v) samples under HIBS and maltose restriction; and (vi) samples supplemented with 200 μM ferrous ammonium sulfate.

**Fig 2:**
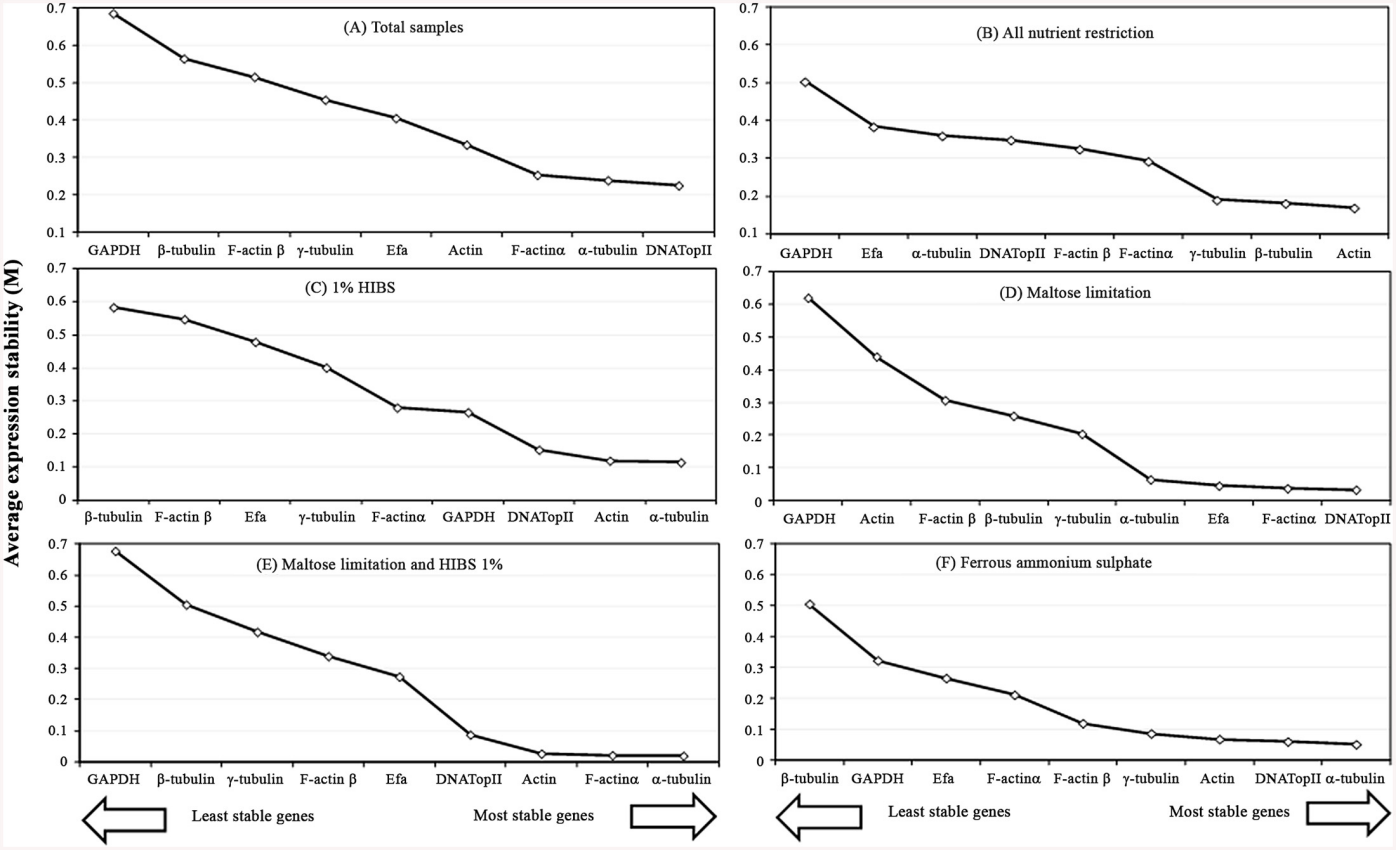
Expression stability values (*M*) and ranking of the candidate reference genes based on geNorm calculation. **(a)** total samples; **(b)** samples under all nutrient restriction; **(c)** samples with 1% HIBS; **(d)** maltose restriction; **(e)** HIBS and maltose restriction; **(f)** samples supplemented with 200 μM ferrous ammonium sulfate. The *M* value and ranking were calculated through a pairwise comparison and stepwise exclusion of the lowest stable gene. Low *M* values correspond to high expression stability.

The average expression stability (*M* value) of the tested genes is based on the average pairwise expression ratio. A low *M* value indicates more stable gene expression, whereas the highest *M* value denotes the least stable reference gene. All candidate reference genes in all six subsets tested showed *M* values lower than the geNorm threshold of 1.5, revealing stability (Fig 2). In the entire set of 15 samples, *DNATopII* (0.225) and *α-tubulin* (0.239) had the lowest *M* values, followed by *F-actin α* and *actin*. Conversely, the *M* value of *GAPDH* was the highest (0.685), suggesting that *DNATopII* and *α-tubulin* present the most stable expression and that *GAPDH* is variably expressed (Fig 2A). The results remained similar in the high-iron experimental subset (Fig 2F), with the lowest *M* values for *α-tubulin* (0.051), *DNATopII* (0.06), and *actin* (0.068), and the highest *M* values for *β-tubulin* (0.505). By contrast, *β-tubulin* (0.182) and *actin* (0.17) were more stable when the complete data sets of the nutrient restriction were grouped together (Fig 2B), and *GAPDH* and *Efa* had highest *M* values, with the lowest expression stability. The *α-tubulin* (0.114) and *actin* (0.119) genes were ranked as the most stable under HIBS restriction (Fig 2C), whereas *F-actin α* (0.038) and *DNATopII* (0.034) were ranked as the most stable under maltose restriction (Fig 2D). For these two last experimental subsets, the unstable genes were *β-tubulin* plus *F-actin α* and *GAPDH* plus *actin*, respectively (Fig 2C and 2D). When there was an association between both nutrient restriction conditions (HIBS and maltose), *F-actin α* (0.022) and *α-tubulin* (0.02) had the most stable expression, in contrast to *GAPDH*, which was variably expressed (Fig 2E).

**Fig 3:**
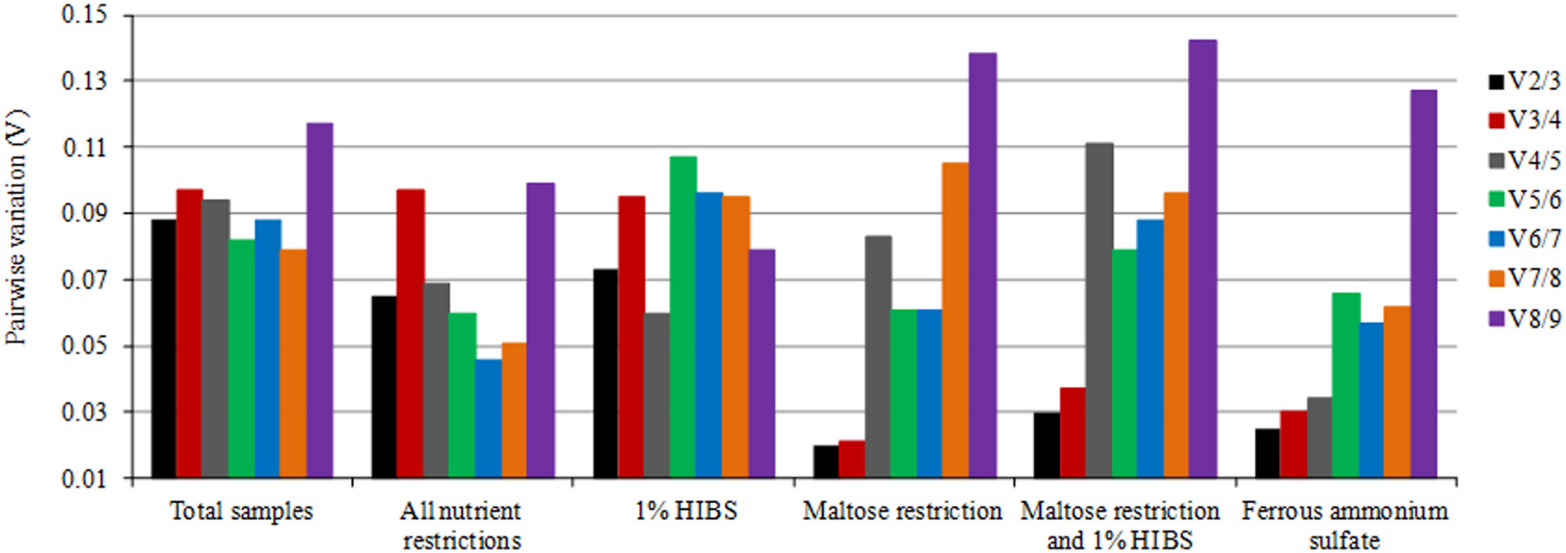
Pairwise variation (*V*) generated by geNorm to identify the optimal number of internal controls. *V* values less than 0.15 indicate that no additional genes are needed to calculated a reliable normalization factor.

The pairwise variations (V*n*/V*n* + 1) were also calculated with geNorm between two sequential ranked normalization genes to determine the minimum number of internal controls needed for an accurate normalization. Analysis of the pairwise variation in all datasets revealed that the optimal number of reference genes in these experimental situations were two, and geNorm *V* was < 0.15 for all subsets compared to a normalization factor based on the 2 or 3 most stable targets (Fig 3).

#### NormFinder analysis

To evaluate the stability of the nine candidate reference genes using the NormFinder algorithm, the samples were grouped into the following two sets: (i) control samples and (ii) all nutrient restriction samples. The expression stability evaluated by the NormFinder program is shown in Table 4. The reference genes with the lowest average expression stability were more stably expressed reference genes. Based on our results, the three most stable reference genes were *DNATopII*, *α-tubulin* and *actin*, and the best combination of two genes were *DNATopII* and *α-tubulin*. When the samples under nutrient restriction were grouped in the same subset, the *α-tubulin*, *actin*, *DNATopII* genes also exhibited the lowest average expression stability; however, the best combination of two genes was *α-tubulin* and *actin* (Table 4).

**Table 4:** Ranking of candidate reference genes in order of expression stability as calculated by NormFinder.

Rank	Total samples			Rank	All nutrient restriction		
	Gene	Stability value	BC_2_G^(+)^		Gene	Stability value	BC_2_G^(+)^
1	*DNATopII* ^(+)^	0.024	0.047	1	*α-tubulin* ^(+)^ ^(a)^	0.021	0.016
2	*α-tubulin* ^(+)^	0.081	0.047	2	*Actin* ^(+)^	0.021	0.016
3	*Actin*	0.089	-	3	*DNATopII*	0.026	-
4	*F-actin α*	0.272	-	4	*F-actin α*	0.065	-
5	*Efa*	0.289	-	5	*Efa*	0.316	-
6	*F-actin β*	0.307	-	6	*F-actin β*	0.403	-
7	*γ-tubulin*	0.362	-	7	*γ-tubulin*	0.449	-
8	*GAPDH*	0.599	-	8	*GAPDH*	0.605	-
9	*β-tubulin*	1.132	-	9	*β-tubulin*	1.348	-

^(+)^ Best combination of two genes and stability value for best combination of two genes

^(a)^ Best gene

### Validation of the selected reference genes in parasites supplemented with ferrous ammonium sulfate

In order to ensure the reliability of the selected reference gene candidates, we analyzed the expression patterns of one iron up-regulated gene (*PFOR*) in trophozoites previously treated with increasing ferrous ammonium sulfate concentrations (100, 200, and 300 μM) at four time points (1, 6, 12, and 24h). For these experiments three *T*. *vaginalis* isolates, one ATCC (30238) and two fresh clinical isolates (TV-LACM6 and TV-LACH4) were used. The *actin* and *DNATopII* genes were selected because of their optimal performance in the geNorm and NormFinder analyses. By contrast, the unstable gene *GAPDH* was also evaluated.

**Fig 4:**
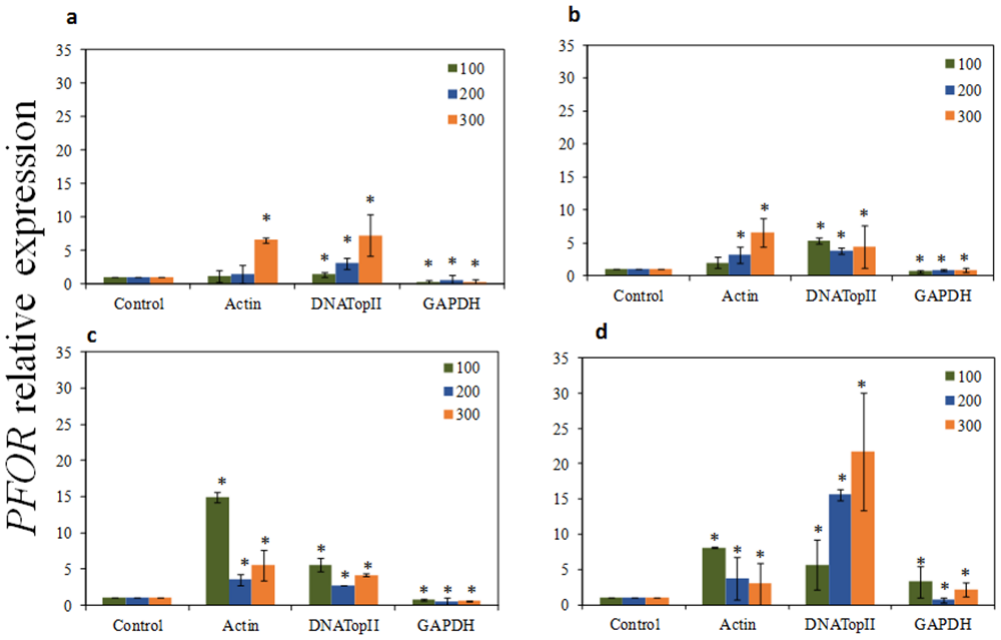
The quantification of *pyruvate-ferredoxin oxidoreductase* (*PFOR*) gene expression in *T*. *vaginalis* ATCC 30238 isolate. The relative expression of *PFOR* gene in *T*. *vaginalis* under ferrous ammonium sulfate (high-iron condition 100, 200, and 300 μM) using *GAPDH*, and *DNATopII* as internal controls, after 1, 6, 12, and 24h of cultivation. (a) The relative expression of *PFOR* gene in *T*. *vaginalis* under high-iron after 1 hour of cultivation; (b) The relative expression of *PFOR* gene in *T*. *vaginalis* under high-iron condition after 6 hours of cultivation; (c) The relative expression of *PFOR* gene in *T*. *vaginalis* under high-iron condition after 12 hours of cultivation; (d) The relative expression of *PFOR* gene in *T*. *vaginalis* under high-iron condition after 24 hours of cultivation. The relative expression levels are depicted as the mean ± SD, calculated from three biological replicate. The relative change in gene expression was analysed using the 2^-ΔΔCt^ method. Statistically significant expression changes were calculated using one-way ANOVA and the level of significance was also determined by the Bonferroni method comparing all groups versus the control. Statistically significance (*P <* 0.001) changes in relative expression are represented with an asterisk.

**Fig 5:**
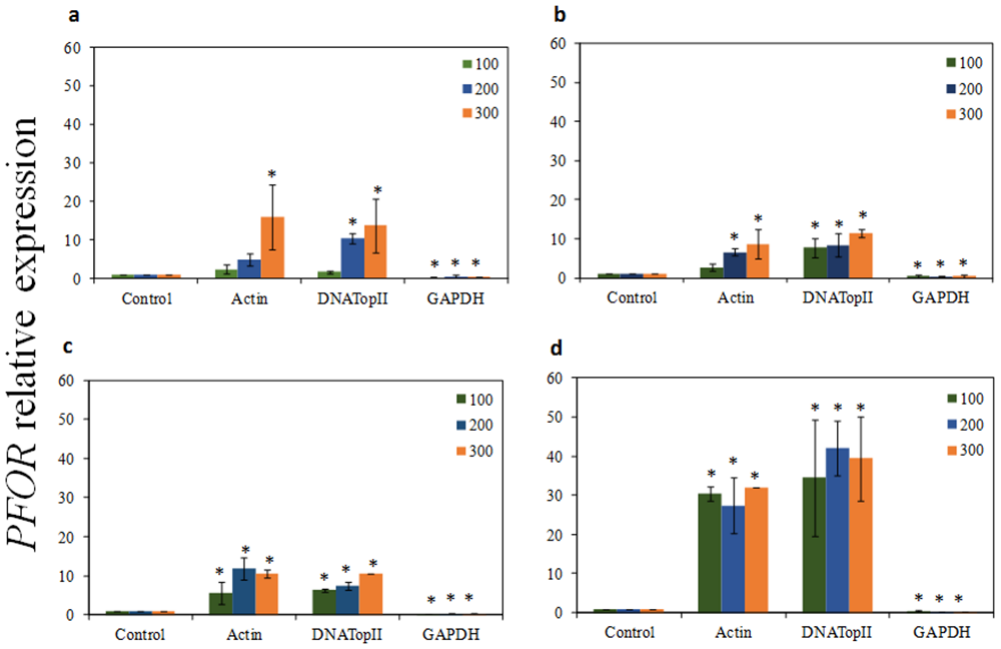
The quantification of *pyruvate-ferredoxin oxidoreductase* (*PFOR*) gene expression in *T*. *vaginalis* TV-LACM6 isolate. The relative expression of *PFOR* gene in *T*. *vaginalis* under ferrous ammonium sulfate (high-iron condition 100, 200, and 300 μM) using *GAPDH*, and *DNATopII* as internal controls, after 1, 6, 12, and 24h of cultivation. (a) The relative expression of *PFOR* gene in *T*. *vaginalis* under high-iron after 1 hour of cultivation; (b) The relative expression of *PFOR* gene in *T*. *vaginalis* under high-iron condition after 6 hours of cultivation; (c) The relative expression of *PFOR* gene in *T*. *vaginalis* under high-iron condition after 12 hours of cultivation; (d) The relative expression of *PFOR* gene in *T*. *vaginalis* under high-iron condition after 24 hours of cultivation. The relative expression levels are depicted as the mean ± SD, calculated from three biological replicate. The relative change in gene expression was analysed using the 2^-ΔΔCt^ method. Statistically significant expression changes were calculated using one-way ANOVA and the level of significance was also determined by the Bonferroni method comparing all groups versus the control. Statistically significance (*P <* 0.001) changes in relative expression are represented with an asterisk.

**Fig 6:**
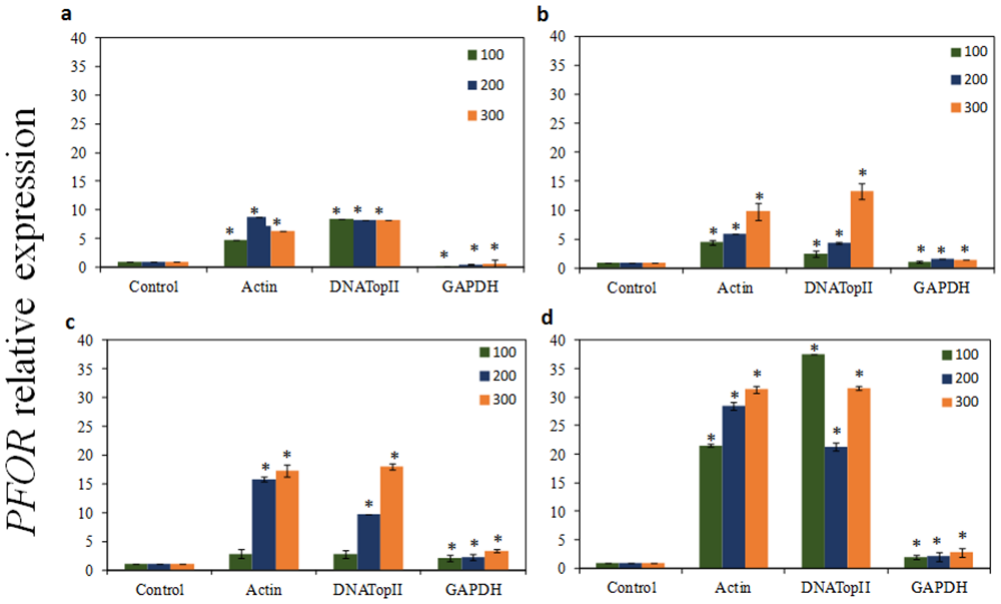
The quantification of *pyruvate-ferredoxin oxidoreductase* (*PFOR*) gene expression in *T*. *vaginalis* TV-LACH4 isolate. The relative expression of *PFOR* gene in *T*. *vaginalis* under ferrous ammonium sulfate (high-iron condition 100, 200, and 300 μM) using *GAPDH*, and *DNATopII* as internal controls, after 1, 6, 12, and 24h of cultivation. (a) The relative expression of *PFOR* gene in *T*. *vaginalis* under high-iron after 1 hour of cultivation; (b) The relative expression of *PFOR* gene in *T*. *vaginalis* under high-iron condition after 6 hours of cultivation; (c) The relative expression of *PFOR* gene in *T*. *vaginalis* under high-iron condition after 12 hours of cultivation; (d) The relative expression of *PFOR* gene in *T*. *vaginalis* under high-iron condition after 24 hours of cultivation. The relative expression levels are depicted as the mean ± SD, calculated from three biological replicate. The relative change in gene expression was analyzed using the 2^-ΔΔCt^ method. Statistically significant expression changes were calculated using one-way ANOVA and the level of significance was also determined by the Bonferroni method comparing all groups versus the control. Statistically significance (*P <* 0.001) changes in relative expression are represented with an asterisk.

As presented in Figs 4, 5 and 6 *PFOR* gene had higher expression pattern among all three *T*. *vaginalis* isolates tested for 1, 6, 12 and 24 h of incubation with ferrous ammonium sulfate when the most stable candidate reference genes (*actin* and *DNATopII*) were used for normalization. Using the other stable reference gene *α-tubulin*, our qRT-PCR analysis also revealed that the *PFOR* transcripts were upregulated on parasites treated with iron at 24h exposition (*P* > 0.05) (S3 Fig). In contrast, when *GAPDH* was used as reference gene, the relative expression of the *PFOR* target varied greatly, showing lower expression level in all experimental conditions tested (*P* > 0.05) than results obtained from normalization with *actin*, *DNATopII*, and *α-tubulin*. Moreover, in Figs 4, 5 and 6 it is also observed that the increase in the *PFOR* gene expression accompanied the increase in iron concentrations. Similarly, the raise in *PFOR* expression was time-dependent, with strongest expression in 24 h. These expression patterns were found among all three *T*. *vaginalis* isolates tested when were used the stable genes as normalizing. However, normalization using *GAPDH* gene resulted in a strong bias, due to significant decrease of the *PFOR* expression despite increase in both iron concentration and time (Figs 4, 5 and 6).

## Discussion

An ideal reference gene should have stable expression level in all tissues, as well as in all organisms culture conditions [13, 15, 16]. However, currently some studies showed that the expression of traditionally used housekeeping genes may diverge when tested under different experimental treatments [17, 18, 43]. Regarding this concern, a set of potential nine reference genes were selected in this study to compare their expression stabilities under different experimental conditions to establish the optimal reference genes for the normalization of qRT-PCR analyses in *T*. *vaginalis*.

The nine-candidate reference genes evaluated here showed a relatively wide range of transcript levels when the raw Ct data was analyzed, and these findings were observed in other studies of gene validation [44]. The *Efa*, *GAPDH*, *α-tubulin* and *actin* genes had low data dispersion suggesting they were most stable genes. These results were consistent with previous studies that evaluated *Efa*, *GAPDH*, *actin*, and *tubulin* as potential reference genes and confirmed that these genes were appropriate as a reference panel for normalizing gene expression data [45, 46]. When average of raw Ct values were analyzed, to estimate expression levels for each candidate reference gene, we found higher expression levels to *Efa*, *GAPDH*, *α-tubulin*, *actin*, and *γ-tubulin*, on the other hand, the *actin*, *GAPDH* and *tubulin* genes had fewer transcripts when evaluated in *Litsea cubeba* [45]. However, there are not previous studies of the validation of reference genes in *T*. *vaginalis* limiting data comparison. Therefore, our contribution is valuable for future studies on *T*. *vaginalis* gene expression analysis.

For an ideal reference gene, a constant Ct value is expected and consequently, low CVs, which ideally should be less than 4.0% [47]. In this study, only three genes had CVs values lower than 4.0% (Table 2). However, the direct comparison of the raw Ct values should be avoided, because they did not result in a precise estimation of the expression stability since the data obtained from the raw Ct values falsely represents the variation in gene expression [48]. Therefore, more powerful methods should be used to evaluate the expression variation of candidate reference genes. In the present study, we used two algorithms, geNorm analysis, which can screen stably expressed genes and determine the optimal number of internal controls, and NormFinder analysis, which calculates the stability value for each gene and ranks the genes.

In geNorm analysis, the nine candidate reference genes in all six sample subsets showed lower *M* values than the geNorm threshold (> 1.5), suggesting that all nine genes tested here are stable. A previous report also described low *M* values for distinct experimental subsets studied, displaying high expression stability [14,46]. However, when the data obtained here were analyzed for each subset, it was possible to determine that there was a difference in the *M* values for all potential reference genes in each sample subsets; therefore, it was possible to classify the genes as most or least stable. *GAPDH* was the most unstable gene in four experimental subsets, as previously shown for chickpea [49]. This is in contrast to a previous report in human keratinocyte cell lines, in which *GAPDH* was the most stable gene [50]. The *β-tubulin* gene, the most used housekeeping gene for both qRT-PCR and RT-PCR in *T*. *vaginalis* studies, was also one of the most unstable genes of the five sample subsets. These results confirm that the selection of reference genes for normalization based on the traditional use as housekeeping genes is an inappropriate approach [17, 18]. *DNATopII*, *α-tubulin*, *F-actin α* and *actin* were the most stable of the nine genes tested and of the six experimental subsets, although some changes in the ranking in each subset occurred. Hence, our findings suggest that the most adequate reference genes were *α-tubulin* and *actin*. These observations confirm the fact that there is no universal reference gene and point to the need of specific optimization of potential reference genes before starting any experimental condition [13].

The optimal number of internal reference genes for normalization was also determined with the geNorm software, which generates the pairwise variation *V* value. The results of all samples subsets showed that the use of two of the most stably expressed reference genes as internal control genes was sufficient because the V_2/3_ was less than 0.15. Therefore, the inclusion of the other genes did not have any effect on the normalization factor.

The ranking of the potential reference genes generated by NormFinder were slightly different from geNorm. The *DNATopII*, *α-tubulin* and *actin* genes were ranked as the most stable in all samples, and the best combination of two genes was *DNATopII* and *α-tubulin*. These two genes were also the most stable genes when all samples were analyzed with the geNorm software. When all nutrient restriction subsets were analyzed with NormFinder, *α-tubulin*, *actin* and *DNATopII* were ranked as the most stable genes, and the best combination was *α-tubulin* and *actin;* however, in the geNorm analyses, the two most stable genes were *actin* and *β-tubulin*.

The most unstable genes were *γ-tubulin*, *GAPDH* and *β-tubulin* for the NormFinder analyses, and *Efa* and *GAPDH* for geNorm. The variation obtained between these two software programs has been described in previous reports on reference genes validation [44,45, 46]. Based on the three analytical tools used, the CV values, NormFinder and geNorm, the most unstable genes were *β-tubulin*, *GAPDH* and *γ-tubulin*. However, both the *β-tubulin* and *GAPDH* genes have been widely used as housekeeping genes in *T*. *vaginalis*, and surprisingly in our study, these two genes were ranked as the most unstable reference genes in different samples subsets. Moreover, when the raw Ct data were evaluated, the *GAPDH* gene had a minor dispersion of data, suggesting stability. This result confirms that in qRT-PCR analyses, the statistical data should be converted to the linear form by the 2^-CT^ calculation and should not be presented as the raw Ct values [48]. Therefore, the use of both *β-tubulin* and *GAPDH* as reference genes should be avoided in qRT-PCR analysis in *T*. *vaginalis*. The combination of *α-tubulin* and *actin* should be used as a reference gene in most of sample subsets because *F-actin α* and *DNATopII* had fewer transcripts and were unsuitable for the normalization of target genes with higher expression levels. Consequently, our results confirm the previously published data, which showed that the stability of gene expression is based on the experimental condition and not only on the species tested [14].

We evaluated the expression levels of the *PFOR* gene in samples previously treated with ferrous ammonium sulfate using *DNATopII*, *α-tubulin*, *actin*, and *GAPDH* as the reference genes. We confirmed that the relative expression profile of *PFOR*, an iron-up-regulated gene, were consistent when using *α-tubulin*, *actin* and *DNATopII*, and the combination of two genes as reference genes. Moreover, a slight difference in *PFOR* expression was observed when *DNATopII*, *α-tubulin*, *actin* were used, although there was no significant difference in *PFOR* expression. In contrast, the normalized expression level showed a reduction in *PFOR* expression when *GAPDH* was used as an internal control, indicating that it is the most unstable gene when parasites are cultivated with ferrous ammonium sulfate.

To reaffirm our results we expanded the number of *T*. *vaginalis* isolates using two fresh clinical isolates. The TV-LACM6 isolate presents remarkable characteristics: high ability to adhere to plastic and to human vaginal epithelial cells, high cytolysis (unpublished data) and the isolate harbors *Mycoplasma hominis* and one *T*. *vaginalis* viruses (TVV) specie (TVV 1) [51]. The isolate TV-LACH4 harbors four distinct TVVs species (TVV 1, 2, 3, and 4) as previously shown by our group [51]. Thus, we evaluated the *PFOR* expression level among all three isolates (ATCC 30238, TV-LACM6, and TV-LACH4) under different ferrous ammonium sulfate concentration in different time points. So, we confirm that the relative expression profile of *PFOR* were in good consistency with increasing ferrous ammonium sulfate concentrations and cultivation times, when *DNATopII* and *actin* were used as reference genes. However, the normalized expression level of the target showed a reduction in expression when using *GAPDH* as internal control, independently of ferrous ammonium sulfate concentration used or time. Consequently, these results are inconsistent with the *PFOR* expression profile, which is a known iron up-regulated gene. Therefore, our findings reinforce that the use of unsuitable internal control may result in data misinterpretation.

Thus, these results reaffirm the reliable use of *DNATopII*, *α-tubulin* and *actin* in combination as a reference in *T*. *vaginalis* studies, since the *PFOR* expression profile were consistence with the increase of ferrous ammonium sulfate concentration in different *T*. *vaginalis* clinical isolates with different genetic and virulence characteristics. Finally, our study confirms the fact that there is no universal reference gene and warns the need of specific optimization of potential reference genes before starting any experimental condition [13,14].

## Conclusion

In the present study, we validated nine candidate reference genes by subjecting the parasites to distinct growth conditions, including HIBS and maltose restriction, as well as supplementation with ferrous ammonium sulfate. The *α-tubulin*, *actin* and *DNATopII* genes exhibited the most stable expression in the majority of samples. Conversely, *GAPDH* and *β-tubulin*, the most used genes in *T*. *vaginalis* studies, were the most unstable. In addition, we suggest that the use of two genes, *α-tubulin* and *actin*, should be sufficient to provide reliable results. To the best of our knowledge, this study is the first systematic exploration of *T*. *vaginalis* to identify optimal reference genes for qRT-PCR normalization under different culture conditions. This is valuable for future research on *T*. *vaginalis* gene expression studies.

## Material and Methods

### Identification of normalizer genes in previous *T*. *vaginalis* studies

A review on the literature was performed to identify the most common genes used as normalizers in *T*. *vaginalis* studies. A search of the PubMed database (http://www.ncbi.nlm.nih.gov/pubmed/) using the keywords “*Trichomonas vaginalis* and qRT-PCR” and “*Trichomonas vaginalis* and RT-PCR” was performed to determine the usual normalizer genes used in quantitative or not quantitative RT-PCR.

### *Trichomonas vaginalis* culture and experimental conditions

*Trichomonas vaginalis* trophozoites, isolate 30238 from the American Type Culture Collection, were cultured axenically *in vitro* in a trypticase-yeast extract maltose (TYM) medium (pH 6.0) supplemented with 10% heat-inactivated bovine serum (HIBS [v/v]) and incubated at 37°C(±0.5) [52]. Organisms exhibiting motility and normal morphology during the logarithmic growth phase were harvested, centrifuged, washed three times with phosphate-buffered saline 1X (PBS) (pH 7.0) and resuspended in fresh TYM medium for subsequent experiments.

Different experimental treatments were used to evaluate the performance of the selected candidate reference genes under four nutritional conditions. For these treatments, 1.0x10^5^ trophozoites/mL were incubated in TYM containing 1.0% HIBS (serum restriction), 273 μM maltose (maltose restriction), 1.0% HIBS and 273 μM maltose (serum plus maltose restriction) and 200 μM ferrous ammonium sulfate (high-iron concentration), for 24 hours at 37°C. The control group represents parasites cultured with TYM containing 27.3 mM maltose and supplemented with 10% HIBS.

To evaluate *PFOR* gene expression in parasites supplemented with ferrous ammonium sulfate, the *T*. *vaginalis* fresh clinical isolates TV-LACM6 and TV-LACH4 were included in this study. These isolates were obtained at Laboratório de Análises Clínicas e Toxicológicas, Faculdade de Farmácia, UFRGS, Brazil, and were registered and stored by cryopreservation at -80C° in the *T*. *vaginalis* isolates bank of our research team (this survey was submitted and approved by the UFRGS Ethical Committee, number 18923). The fresh clinical isolates were grown under the same conditions as described above. All treatments were performed on three different days.

### Primer designs

The Primer3 designing software (http://www.bioinformatics.nl/cgi-bin/primer3plus/primer3plus.cgi) was used to design primer pairs using the following criteria: product size between 100 and 200 bp, Tm of approximately 60°C, GC content of 40–60% and primers length of 18–22 bp. The generated primer pair for each gene was then aligned against the *T*. *vaginalis* genome to confirm its specificity *in silico*. The primer pairs were also evaluated for primer dimer formation using multiple primer analyzers (http://www.thermoscientificbio.com/webtools/multipleprimer/). The forward and reverse primers were intentionally targeted to the adjoining exons, which were separated by an intron. The gene ID, primer sequence and gene symbol are shown in Table 1 for all nucleotide sequences assessed here.

### PCR efficiency and specificity

A 10-fold serial dilution consisting of five samples starting from 100 ng genomic DNA was used to construct standard curves to determine the PCR amplification efficiencies (E) for each candidate reference gene. The DNA was extracted from ~4.0 x10^6^ trophozoites/mL using the AxyPrep Multisource Genomic DNA Miniprep Kit (BioScience, Inc) according to the manufacturer's recommendations. The PCR amplification specificity for each candidate reference gene was assessed by melting curve analyses and agarose gel electrophoresis. Three technical replicates from three biological replicates were analyzed.

### RNA extraction

The total RNA was extracted from ~4.0 x10^6^ trophozoites/mL using TriZol^TM^ following the manufacturer’s instructions and was stored at -80°C until use. The integrity of the RNA samples was determined by 2.0% agarose gel electrophoresis and well-defined bands that confirmed the absence of nucleic acid degradation. The quantity and purity of the RNA were determined using spectrophotometric method, and only the high-quality samples, in which the A_260_/A_280_ was 1.8 and A_260_/A_230_ was 2.0, were used for subsequent qRT-PCR analyses. The total RNA samples were pretreated with DNAse, to ensure that there was no contamination with genomic DNA in the qRT-PCR analysis.

### Real-time PCR analyses and quantitative reverse transcriptase PCR (qRT-PCR) amplifications

To determine the primer standard curve, real-time PCR reactions were performed in 0.1-mL microtubes using the Qiagen real-time PCR system, Rotor-Gene Q and Rotor-Gene^TM^ SYBR^TM^ Green RT-PCR kit (Qiagen^TM^). Each PCR reaction contained 6 μL of 2x Rotor-Gene SYBR Green PCR Master Mix, 100 nM or 200 nM (Table 1) of each primer and 2 μL of genomic DNA template in a total volume of 12 μL. The annealing temperatures and primer concentration were selected for the highest amplification, best product specificity and no primer dimer amplification based on the melting curve analyses. The cycling conditions were as follows: initial enzyme activation step at 95°C for 10 min, followed by 35 cycles of denaturation at 95°C for 15 s and annealing and extension at 63°C or 64°C (Table 1) for 30 s, with fluorescence data collection recording in this step. Melting curve analyses were performed by raising the temperature at the end of each run in by 1°C per 5 s from 63°C or 64°C to 95°C. No DNA template controls were also included for each primer pair as a negative control. For quantitative reverse transcription, 100 ng of RNA and 0.125 μL Rotor-Gene RT Mix were added to each reaction. The qRT-PCR cycling was an initial step at 55°C for 10 min, followed by polymerase activation and PCR cycling as described above. Parallel reactions without both RNA template and transcriptase reverse enzyme were used as the negative control. Three biological replicate samples were analyzed in three technical replicates for each experimental condition.

### Expression level of the selected candidate reference genes

The values of the cycle threshold (Ct) for each reaction were calculated by the Rotor-Gene Q series software 2.1.0 and these values were used to determine the average Ct for each sample. At least two of three technical replicates were considered, and any replicate showing non-specific products in the melting curve analyses was excluded from the average Ct calculation. The Ct averages from the technical replicates obtained from three biological replicates was used as the raw Ct data.

### Data analyses

To determine the best reference genes among the different culture conditions, two independent statistical algorithms were used, geNorm and NormFinder [15,21]. For the geNorm analysis, the raw data Ct values were entered into the geNorm of the qBase^PLUS^ V 2.4 software [53], and for the NormFinder, the raw data were transformed to the relative quantities using the delta-Ct method Q = 2^-ΔCt^ [48]. NormFinder was used to calculate the stability value of the reference genes based on their intra- and inter-expression variation, and those that exhibited lower average expression stability values were regarded as more stably expressed reference genes. The geNorm software was used to calculate the expression stability value (*M*) and the mean pairwise variation (*V* value, *V*_*n/n+1*_) between all of the tested genes. The threshold of *V* < 0.15 was used in this study [15,21].

### Validation of the selected reference genes

To validate the selected reference genes, the two most stable genes and the most unstable gene were used to analyze the relative expression levels of pyruvate-ferredoxin oxidoreductase (PFOR), which is an iron-up-regulated gene. For this assay, trichomonads from fresh clinical isolates, TV-LACM6 and TV-LACH4 were cultured in increasing iron concentrations (100, 200, and 300 μM) and the total RNA was extracted in different times: 1, 6, 12, and 24 hours. The total RNA was pre-treated with DNase I (Invitrogen^TM^) following the manufacturer's instructions prior to the qRT-PCR. The *PFOR* primers were as follows: *PFOR*_AP120:_ forward 5’CTCGTTTGGGGTGCTACATT3’ and reverse 5’TCCTGATCCCAAACCTTGAG3’ (TVAG_198110; 239 bp). Three biological replicates were performed and the transcripts of the *PFOR* genes were quantified by qRT-PCR, and three technical replicates were performed for each sample. The relative change in gene expression was analyzed using the 2^-ΔΔCt^ method [48]. The *actin*, *α-tubulin* and *DNATopII* genes identified in this study as reference genes were used for normalization. The *GAPDH* gene was tested as the least stable reference gene. Statistically significant expression changes were calculated using one-way ANOVA. The level of significance was also determined by the Bonferroni method comparing all groups versus the control (*P <* 0.001).

## Supporting Information

S1 Fig Phylogenetic trees constructed from the candidate reference genes sequences. (A) *Actin*; (B) *F-Actin β*; (C) *α-tubulin*; (D) *β-Tubulin*; (E) *γ-tubulin*; (F) *GAPDH*; (*) the asterisks represent the sequences used in this study.
(TIF)

S2 Fig Confirmation of primer specificity and amplicon size. (A) Melting curve of nine candidate reference genes. (B) Agarose gel (2.0%) showing the specific RT-qPCR product of the expected size for each gene. M represents a 2080 bp DNA marker.
(TIF)

S3 Fig The quantification of *pyruvate-ferredoxin oxidoreductase* (*PFOR*) gene expression. The relative expression of *PFOR* gene in *T*. *vaginalis* under ferrous ammonium sulfate (high-iron condition 200 μM) using *GAPDH*, *α-tubulin*, *actin*, and *DNATopII* as internal controls, after 24h of cultivation. The relative expression levels are depicted as the mean ± SD, calculated from three biological replicate. The relative change in gene expression was analyzed using the 2^-ΔΔCt^ method. Statistically significant expression changes were calculated using one-way ANOVA and the level of significance was also determined by the Bonferroni method comparing all groups versus the control. Statistically significance (*P <* 0.001) changes in relative expression are represented with an asterisk.
(TIF)
