# Supplementary figures and images for: Optimal Reference Genes for Gene Expression Normalization in Trichomonas vaginalis
Source: PLoS One. 2015 Sep 22;10(9):e0138331. doi: 10.1371/journal.pone.0138331 (PMC4579074; doi:10.1371/journal.pone.0138331)

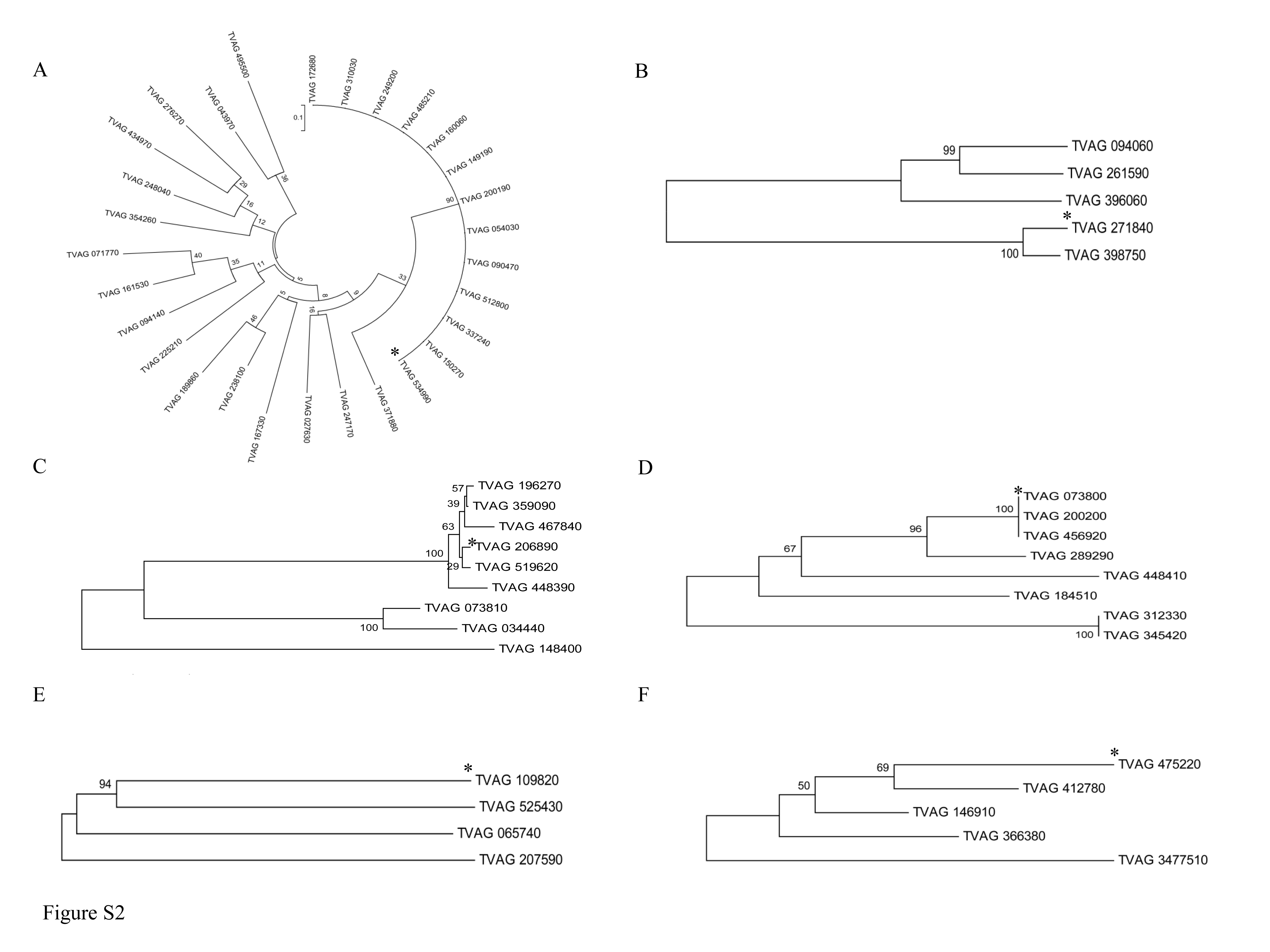

Supplement: S1 Fig — (A) Actin; (B) F-Actin β; (C) α-tubulin; (D) β-Tubulin; (E) γ-tubulin; (F) GAPDH; (*) the asterisks represent the sequences used in this study. (TIF) [file pone.0138331.s001.tif]

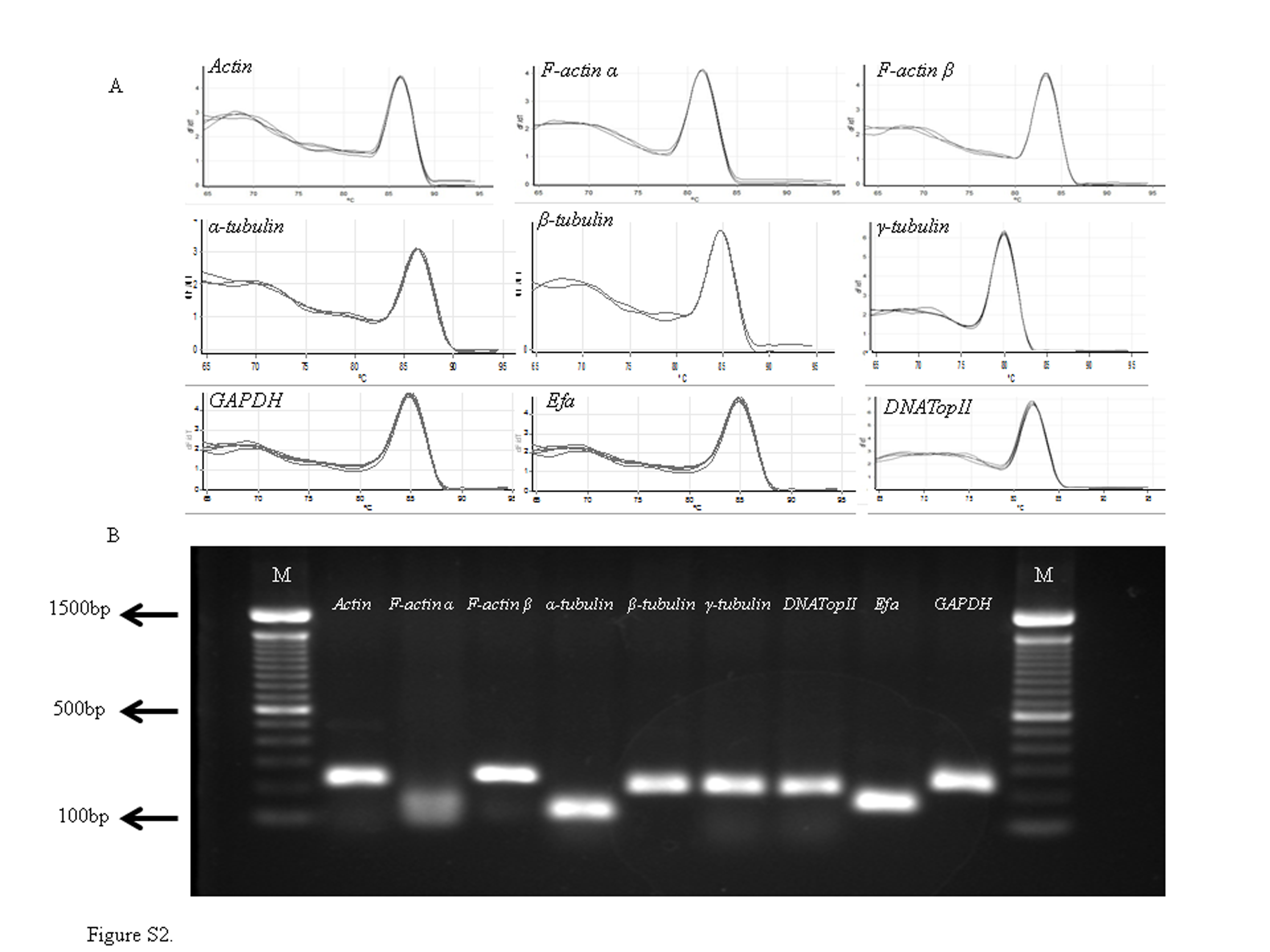

Supplement: S2 Fig — (A) Melting curve of nine candidate reference genes. (B) Agarose gel (2.0%) showing the specific RT-qPCR product of the expected size for each gene. M represents a 2080 bp DNA marker. (TIF) [file pone.0138331.s002.tif]

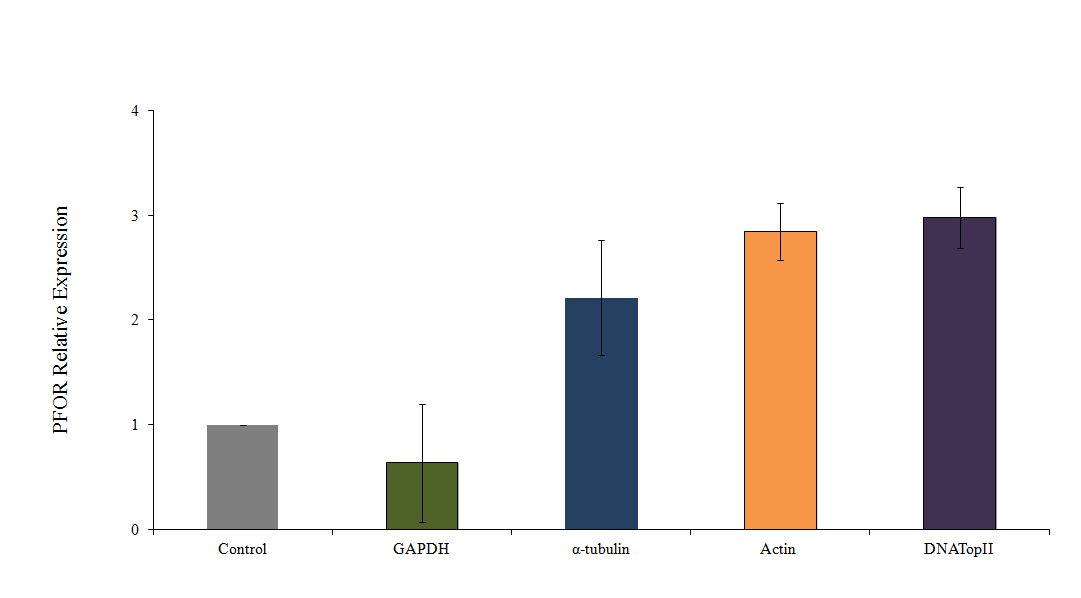

Supplement: S3 Fig — The relative expression of PFOR gene in T. vaginalis under ferrous ammonium sulfate (high-iron condition 200 μM) using GAPDH, α-tubulin, actin, and DNATopII as internal controls, after 24h of cultivation. The relative expression levels are depicted as the mean ± SD, calculated from three biological replicate. The relative change in gene expression was analyzed using the 2-ΔΔCt method. Statistically significant expression changes were calculated using one-way ANOVA and the level of significance was also determined by the Bonferroni method comparing all groups versus the control. Statistically significance (P < 0.001) changes in relative expression are represented with an asterisk. (TIF) [file pone.0138331.s003.tif]
